# Claudin18.2 is a novel molecular biomarker for tumor-targeted immunotherapy

**DOI:** 10.1186/s40364-022-00385-1

**Published:** 2022-05-31

**Authors:** Weijie Cao, Haizhou Xing, Yingmei Li, Wenliang Tian, Yongping Song, Zhongxing Jiang, Jifeng Yu

**Affiliations:** Department of Hematology, The First Affiliated Hospital of Zhengzhou University, Zhengzhou, 450052 Henan China

**Keywords:** Claudin18.2, Molecular biomarker, Immunotherapy, Monoclonal antibody (mAb), Bispecific antibody (BsAb), Chimeric antigen receptor T (CAR-T) cells, Antibody–drug conjugates (ADCs), Clinical trial

## Abstract

The claudin18.2 (CLDN18.2) protein, an isoform of claudin18, a member of the tight junction protein family, is a highly selective biomarker with limited expression in normal tissues and often abnormal expression during the occurrence and development of various primary malignant tumors, such as gastric cancer/gastroesophageal junction (GC/GEJ) cancer, breast cancer, colon cancer, liver cancer, head and neck cancer, bronchial cancer and non-small-cell lung cancer. CLDN18.2 participates in the proliferation, differentiation and migration of tumor cells. Recent studies have identified CLDN18.2 expression as a potential specific marker for the diagnosis and treatment of these tumors. With its specific expression pattern, CLDN18.2 has become a unique molecule for targeted therapy in different cancers, especially in GC; for example, agents such as zolbetuximab (claudiximab, IMAB362), a monoclonal antibody (mAb) against CLDN18.2, have been developed. In this review, we outline recent advances in the development of immunotherapy strategies targeting CLDN18.2, including monoclonal antibodies (mAbs), bispecific antibodies (BsAbs), chimeric antigen receptor T (CAR-T) cells redirected to target CLDN18.2, and antibody–drug conjugates (ADCs).

## Introduction

**Fig. 1:**
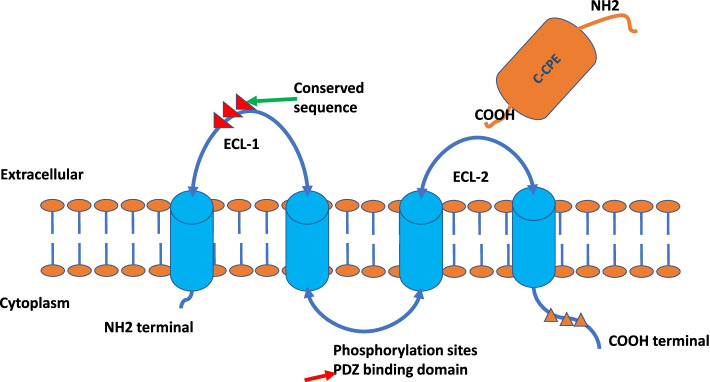
The depiction of Claudin protein structures. Four transmembrane domains (TMDs) with two extracellular loops (ECL), ECL-1 and ECL-2. The ECL-1 contains the highest conserved sequence and the C-CPE C-terminal binds to the ECL-2. Phosphorylation sites locate at the C-terminal of the cytoplasmic site

Claudins (CLDNs) are a family of proteins and are important components of tight junctions (TJs) [1], which form a paracellular barrier to control the flow of molecules between cells. CLDNs have transmembrane domains and an N-terminus and a C-terminus in the cytoplasm (Fig. 1). Different CLDNs are expressed in different tissues, such as gastric, pancreatic and lung tissues, and their altered tissue function has been linked to the formation of cancers in the respective tissue [2, 3]. The expression of different CLDNs has been shown to have prognostic significance in different cancers, for example, claudin-1 has been linked with prognosis in colon cancer [4], claudin-18 has been linked with prognosis in gastric cancer (GC) [5], and claudin-10 has been linked with prognosis in hepatocellular carcinoma [6].

Claudin18.2 (CLDN18.2) is a highly selective marker protein that is exclusively expressed in differentiated gastric mucosal membrane epithelial cells, has highly limited expression in normal healthy tissues and is not expressed in undifferentiated gastric stem cells [7]. In normal healthy tissue, CLDN18.2 is typically present in the tight junctions of gastric mucosal cells [8–11], and CLDN18.2 maintains the barrier function of gastric mucosa and prevents the leakage of H+ in gastric acid through paracellular pathways [12]. That CLDN18.2 overexpression has been identified in several other types of cancers, including pancreatic cancer (PC) [13, 14], esophageal cancer, ovarian adenocarcinoma and lung cancers, also indicates the potential of CLDN18.2 for the diagnosis and treatment of other tumors [10]. These findings not only provide new insights into the tissue expression and regulation of this tissue-specific tight junction protein but also support CLDN18.2 as a candidate target for the development of therapeutic antibodies [10, 13].

However, CLDN18.2 expression is frequently aberrant during the occurrence and development of a variety of malignant tumors. Upon malignant transformation of gastric epithelial tissue, perturbations in cell polarity lead to cell surface exposure of CLDN18.2 epitopes, and CLDN18.2 becomes highly, selectively and stably expressed in specific tumor tissues [15]. The CLDN18.2 protein participates in the proliferation, differentiation and migration of tumor cells. With its specific expression pattern, CLDN18.2 has become a unique molecular target for targeted therapy in different cancers [16], especially in GC. For example, zolbetuximab (claudiximab, IMAB362), a monoclonal antibody (mAb) against CLDN18.2, has been developed [7] for the treatment of GC.

### The gene structure and protein expression of claudins

**Fig. 2:**
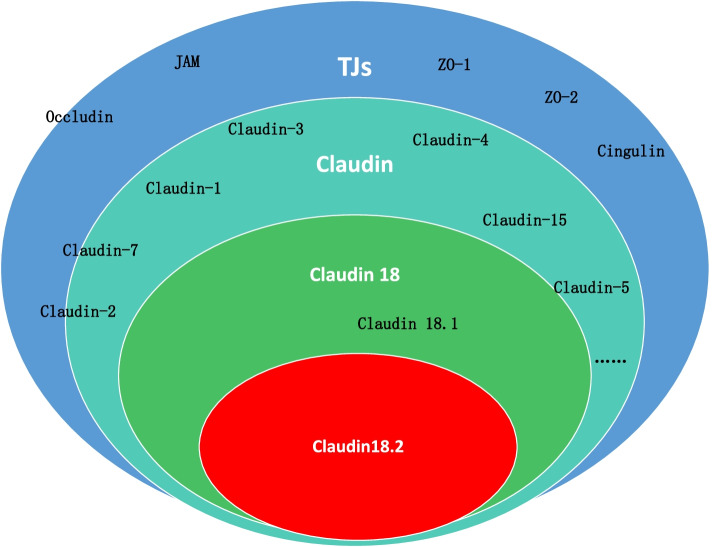
The classification of the TJs and claudins

**Fig. 3:**
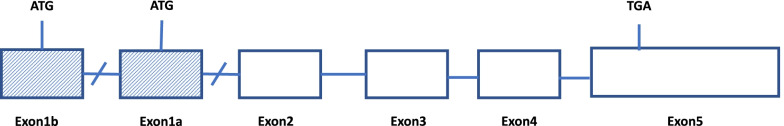
The depiction of genomic organization of the human Claudin18 gene locus on chromosome 3q22, Claudin18.1 and CLaudin18.2 result from alternative splicing of the first exon

CLDNs are a family of TJ proteins involved in the regulation of the permeability, barrier function, and polarity of epithelial layers [17–19]. Thus far, 27 isoforms of the CLDN family have been defined in mammals (Fig. 2) [20]. The human CLDN18 gene locus on chromosome 3q22 covers approximately 35 kb and is organized into 6 exons and 5 introns (Fig. 3) [9]. Alternative splicing of exons 1a and 1b forms the two isoforms CLDN18.1 and CLDN18.2 [9]. CLDNs are tetraspanin proteins ranging from 20 to 27 kDa composed of four transmembrane domains (TMDs), an N-terminus and a C-terminus in the cytoplasm and two extracellular loops that span the TMDs (Fig. 1) [7, 20]. Alteration of the function of any of the 27 CLDN isoforms in distinct human organs may play a critical role in carcinogenesis in the respective tissues, affecting stages from metaplasia to progression to metastasis [21–23]. Among these proteins, CLDN1–5, CLDN7–12, CLDN16 and CLDN18 are expressed in normal gastric mucosa. In particular, expression of isoform 2 of claudin-18 (CLDN18.2) is confined to differentiated and stem gastric epithelial cells, where it controls paracellular permeability to Na^+^ and H^+^ ions. Epitopes of CLDN18.2 within the TJ supramolecular complex in normal tissues are largely inaccessible by intravenously administered antibodies [8–10]. However, the disturbed cell polarity associated with carcinogenesis leads to exposure of CLDN18.2 epitopes, making them available for binding of target monoclonal antibodies [8–11]. CLDN18.2 expression is maintained in GC and gastric metastases [10, 22, 24]. These features make CLDN18.2 a unique biomarker and target for targeted immune therapies, especially in GC [25], in which monoclonal antibodies specifically binding to CLDN18.2 with the capability of reduced off-target effects have been developed [26].

**Fig. 4:**
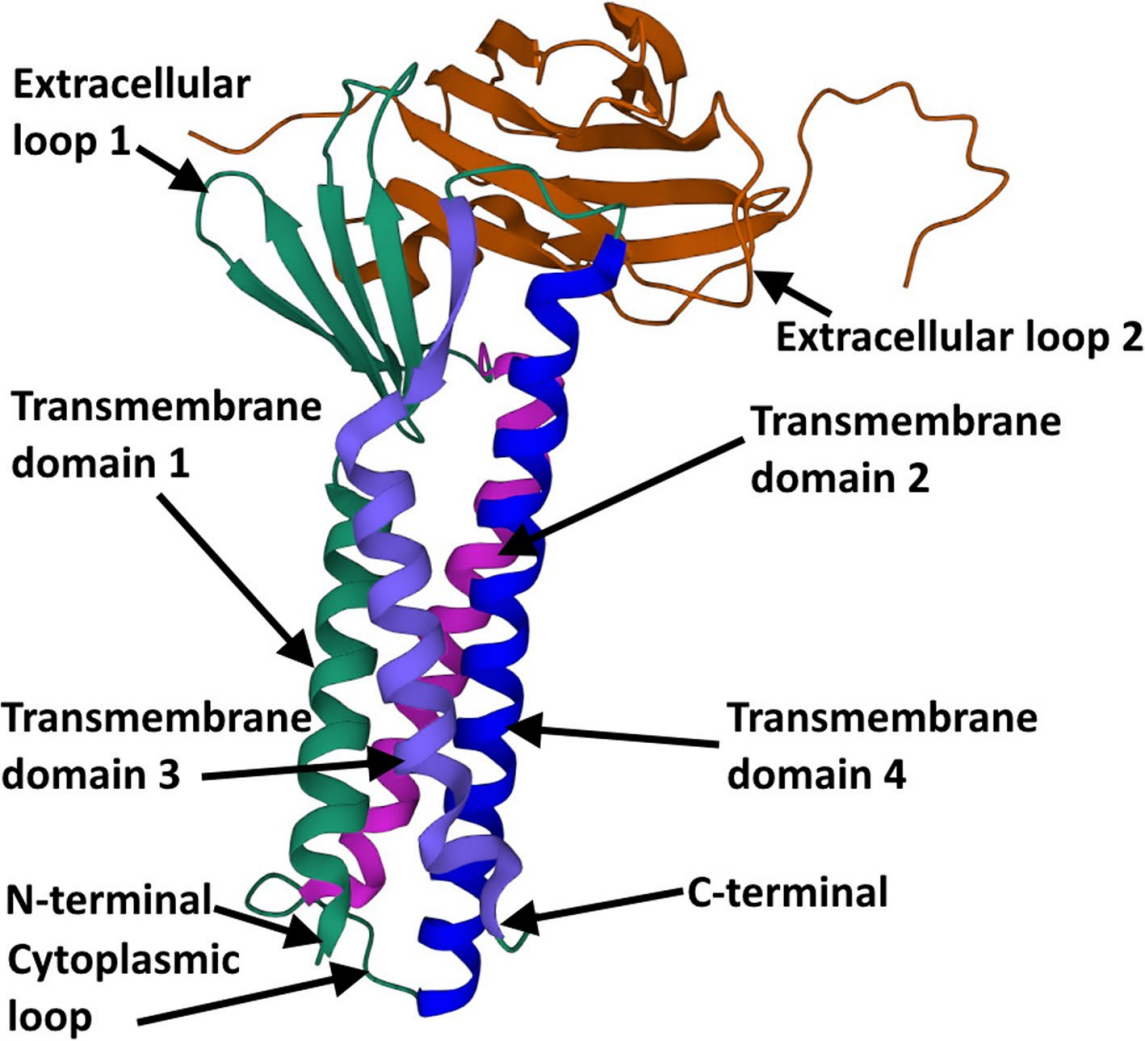
The crystal structure of the human Claudin4 gives the basic illustration of the Claudins structure, which contains the same main structures for all 27 isoforms of Claudins. 10.1073/pnas.2024651118, https://www.rcsb.org/structure/7kp4

After the crystal structure of mouse CLDN-15 revealed the first snapshot of a CLDN monomer at atomic resolution [27], a new model based on the crystallographic arrangement of CLDN-15 and the results of cysteine cross-linking experiments was established from mutants of CLDN-15 and CLDN-2 [28–30]. Furthermore, strand dimensions were observed based on freeze-fracture electron microscopy images, and the amino acid structure was revealed by crystallography (Fig. 4) [27, 31–35]. The protein structure of mouse CLDN-19 and human CLDN-4 with the C-terminal fragment of the bacterial *Clostridium perfringens* enterotoxin (C-CPE) was revealed and had almost the same structural framework as mouse CLDN-15 [32, 33, 35]. The phosphorylation of the C-terminal tail of CLDNs interacts with several major kinases; for example, CLDN3 is phosphorylated by protein kinase A to induce disruption of TJs [36], and CLDN5 is phosphorylated by protein kinases C, α and ζ [37, 38]. The phosphorylation of CLDNs may downregulate TJ strength [36, 38, 39]. In addition, orthologous proteins of the two isoforms of human CLDN18 protein have been found in all species, including macaques, mice, rats, dogs, and rabbits, via sequence similarity searches and genome alignments [27, 31–34, 40–43]. These findings indicate the conservative characteristics of CLDN18.

### The CLDN18 signaling pathway

A mechanistic study demonstrated that CLDN18 regulates cell lineage differentiation and cellular signaling in the mouse stomach and that CLDN18-knockout mice develop intraepithelial neoplasia, indicating that loss of the TJ protein CLDN18 promotes progressive neoplasia development in the mouse stomach [44]. Another study in human PC cells revealed that CLDN18 is primarily regulated at the transcriptional level via specific protein kinase C signaling pathways and modification of DNA methylation [45]. A recent study suggested that the SPAK-p38 MAPK signaling pathway modulates CLDN18 and the barrier function of the alveolar epithelium after hyperoxic exposure [46]. Furthermore, a clinical pathology study showed that most advanced gastric tumors with ras homolog gene family member A (RHOA) mutation have the diffuse-type GC growth pattern, but this mutation had limited prognostic impact in isolation [47]. Another study revealed that CLDN18 coupled with modulation of epidermal growth factor receptor (EGFR) / extracellular signal-regulated kinase (ERK) signaling contributes to the malignant potential of bile duct cancer [48]. Thus, CLDN18.2, as a unique molecular biomarker, may be used for targeted therapy in other clinical applications in addition to GC treatment.

A recent meta-analysis using miRNA and gene expression profiles downloaded from the Gene Expression Omnibus database identified differentially expressed genes and miRNAs between an idiopathic pulmonary fibrosis (IPF) group and a normal group, and CLDN18 was identified as a potential IPF biomarker. However, the specific roles of these genes and miRNAs in IPF need further investigation [49]. Another study to analyze the expression, genetic alterations, and prognostic role of *CLDN18* using public data from The Cancer Genome Atlas (TCGA), Gene Expression Omnibus (GEO), and Human Protein Atlas (HPA) databases demonstrated that the expression of *CLDN18* was restricted to the lung and stomach in normal tissues and was significantly downregulated in GC but was ectopically overexpressed in some other cancer types. There was no correlation between the mRNA expression of *CLDN18* and the clinicopathology of GC, although the expression was higher in the Epstein–Barr virus (EBV)-positive subgroup than in the other subgroups. Genetic alteration of *CLDN18* was not a common event in GC; the main alteration was gene fusion with ARHGAP26. *CLDN18* expression did not predict the overall survival (OS) of GC patients [50]. A preclinical study showed that *CLDN18* knockout has no effect on tight junction permeability in the stomach but rather affects anion permeability, suggesting that *CLDN18* loss alters transcellular chloride flux but not TJ ion selectivity in gastric epithelial cells [51].

### The expression of claudin18.2 in different tumors

CLDN 18.2 is a CD20-like differentiated protein. Although its expression is very limited in normal tissues, it is often abnormally overexpressed during the occurrence and development of various primary malignant tumors. CLDN18.2 was initially found to be consistently and stably expressed in GC tissues [24]. However, subsequent studies showed that it is also activated and overexpressed in many primary cancers, such as breast cancer, colon cancer, liver cancer, head and neck cancer, bronchial cancer and non-small-cell lung cancer, and especially in digestive system malignancies, including GC, PC, and esophageal cancer [10, 52]. In another study [53], normal pancreatic tissues with different structures and cell types, including ductal cells, acinar cells, vesicle cells, endocrine glands, exocrine glands, α cells, β cells, δ cells, ε cells and pancreatic polypeptide-producing cells, were comprehensively examined, and CLDN18.2 was not detected in any of these components. However, of 174 cases of primary pancreatic ductal adenocarcinoma, the most frequent type of pancreatic neoplasm, 59.2% of cases had ≥1% cells with CLDN18.2 positive expression, and most of the cells had > 2+ CLDN18.2 staining intensity. In patients with pancreatic neuroendocrine tumors, the positive rate of CLDN18.2 expression was relatively low; only 20% of patients had CLDN18.2 positivity, but the staining intensity in all patients was > 2+. Though this disease is rare, the development of a precision drug for the disease is still of interest given the young age of patients. An additional study that employed immunohistochemistry (IHC) staining of samples from 105 patients with advanced gastric signet-ring cell carcinoma (SRCC) showed that 95.2% of the samples were positive. Moderate-to-strong CLDN18.2 expression was observed in 64.8% (68/105) of the samples. In particular, 21.0% (22/105) of the samples had positive staining in more than 90% of the tumor cells [54]. Another study used a tissue microarray to detect CLDN18.2 IHC staining in samples from patients with non-small-cell lung cancer (NSCLC) [55] and revealed distinct membranous positivity of CLDN18.2 (3.7%) in nonoverlapping subgroups of adenocarcinomas and large-cell carcinomas. CLDN18.2 expression in normal lung tissue and bronchial mucosal epithelium was negative. The group of CLDN18.2-positive tumors was enriched in slowly proliferating, thyroid transcription factor 1 (TTF-1)-negative adenocarcinomas, suggesting that isoform-specific CLDN18.2 expression may be indicative of that specific subtype. Another study found that the expression of CLDN18.2 in the damaged squamous epithelium of Barrett’s esophagus was significantly increased, indicating that abnormal CLDN18.2 activation may be an early event of esophageal cancer [56]. TTF-1 is a regulator of surfactant protein expression, which is mainly expressed in lung adenocarcinoma and considered as one of the major diagnostic markers for lung adenocarcinoma [57] . However, the clinical significance of the relationship between CLDN18.2 and TTF-1 expression needs to be further explored.

The expression of CLDN18.2 is not limited to primary lesions; CLDN18.2 is also highly expressed in metastases and may be involved in malignant tumor cell proliferation and chemotaxis. In a study of patients with PC metastasis, the positive rates of CLDN18.2 expression in the lymph node metastasis group, liver metastasis group and nonmetastasis group were 70, 66 and 59%, respectively [53]. The expression level of CLDN18.2 was significantly higher in metastatic lymph nodes than in primary tumors and was negatively correlated with the prognosis of lymph node-positive patients. Lymph node positivity is an independent factor predicting poor prognosis of PC. At present, the mechanism by which CLDN18.2 promotes lymph node metastasis and distant metastasis of malignant tumors remains unknown, but it may be related to its abnormal expression changing the structure and function of tight junctions between malignant tumor cells [58].

Some studies have explored the mechanism by which CLDN18.2 expression is regulated. One study demonstrated that CLDN18.2 expression is mainly regulated by the CLDN18.2 promoter, which includes a binding site for the transcription factor cyclic AMP-responsive element binding protein (CREB), a protein that is known to regulate the activation of human CLDN18.2 [9]. Another study showed that the activation of CLDN18.2 depends on the binding of the transcription factor CREB to a hypomethylated CpG island. CREB phosphorylation alone is not a reliable predictor of target gene activation, and additional CREB regulatory partners are required for recruitment of the transcriptional apparatus to the promoter. After cAMP activates protein kinase A (PKA) in tumor cells, activated PKA enters the nucleus and induces phosphorylation to activate CREB. The transcriptional activity of CREB increases, and activated CREB binds with the CpG island promoter, resulting in abnormal activation and overexpression of CLDN18.2 [59, 60]. In human PC cells, CLDN18.2 is primarily regulated at the transcriptional level via specific PKC signaling pathways and modified by DNA methylation [45]. A recent study revealed downregulation of miR-767-3p and upregulation of *CLDN18.2* in lung adenocarcinoma tissue and cell lines. There was a negative correlation between the expression of miR-767-3p and CLDN18.2 in lung adenocarcinoma, and miR-767-3p was found to modulate the expression of CLDN18.2 by binding its 3′-untranslated region (3′-UTR). In another study, knockdown of CLDN18.2 resulted in a decrease in the growth, migration, and invasion of lung adenocarcinoma cells [61]. However, that study did not further reveal the regulatory effect of miR-767-3p on CLDN18.2 expression.

CLDN18.2 expression and its correlation with various clinicopathological factors in GC were explored using appropriate tissue specimens in a Caucasian cohort. The expression of CLDN18.2 was studied in 481 GCs by immunohistochemistry analysis of whole tissue sections. Immunostained GCs were evaluated using the histoscore (H-score) and subsequently divided into two groups: tumors showing any or no expression. Scoring of each tumor was assessed by determining the H-score, and subsequently, a semiquantitative approach combining both the immunostaining intensity and the percentage of positive cells of the tumor was employed. The IHC score reflected strong (3+), intermediate (2+), weak (1+), or lack of (0) membranous staining of CLDN18.2 in tumor cells. An IHC score of 3+ was given if strong staining was present in the cells surrounding tumor cells. CLDN18.2 expression was found in 203 GCs (42.2%). Of these tumors, 71 (14.8%) showed weak immunostaining. CLDN18.2 expression correlated with mucin phenotype, EBV status, and integrin αvβ5, EpCAM extracellular domain EpEX, and lysozyme levels. No correlation of CLDN18.2 expression with survival, Lauren phenotype, or any other clinicopathological patient characteristic was found. The discovery of the novel correlations of CLDN18.2 expression with αvβ5, EpEX, and lysozyme levels may pave the way for further investigations regarding the role of tight junction proteins in GC progression [62].

Another study to evaluate the clinical relevance of CLDN18.2 expression in GC including 367 patients from Korea showed that CLDN18.2 expression was observed in 273 patients (74.4%), and 108 (29.4%) patients were classified as having positive CLDN18.2 expression with 11–100% of cells having moderate to strong staining intensity (2 or 3). The positive expression rates were higher in diffuse-type and HER2-positive tumors. CLDN18.2 expression was not correlated with age, sex, tumor location, stage, or survival outcomes [63].

A study on CLDN18.2 expression in the primary tumors and lymph node (LN) metastases of Japanese patients with GC showed that among 263 samples analyzed (134 primary gastric tumors and corresponding LN metastases; 128 primary tumors only; one LN metastasis only), CLDN18.2 was detected in 87% (*n* = 228/262) of all primary tumors and 80% (*n* = 108/135) of LN metastases. Moderate-to-strong CLDN18.2 expression (≥2+ membrane staining intensity in ≥40% of tumor cells [FAST eligibility criterion]) was observed in 52% (*n* = 135/262) of primary tumors and 45% (*n* = 61/135) of (LN) metastases. CLDN18.2 expression was significantly higher in GCs of the diffuse histological subtype per Lauren classification and in high-grade tumors [64].

In the MONO study, a multicenter, phase IIa study of zolbetuximab as a single agent in patients with recurrent or refractory advanced adenocarcinoma of the stomach or lower esophagus, patients with advanced gastric, gastro-esophageal junction (GEJ) or esophageal adenocarcinomas with moderate-to-strong CLDN18.2 expression in > 50% of tumor cells were eligible to receive zolbetuximab treatment. Among the 268 patients screened for CLDN18.2 expression, only 54 (20%) showed CLDN18.2 expression in > 50% of tumor cells [65]. Furthermore, in the FAST study, a randomized phase II study of zolbetuximab (IMAB362) plus EOX versus EOX alone for first-line treatment of advanced CLDN18.2-positive GC and gastro-esophageal adenocarcinoma, CLDN18.2 positivity was defined as moderate (2+) or strong CLDN18 staining (3+) in at least 40% of the tumor cells. Among the 686 patients assessed for CLDN18.2 expression, 334 (48.7%) patients were positive for CLDN18.2 [11].

Regardless, due to differences in IHC detection methods, positive criteria thresholds and patient populations, CLDN18.2 positive rates vary among trials. Of the patients included in the FAST and MONO trials, 49 and 45% were white, and 36 and 24% of these populations, respectively, had high expression levels [11, 65]. To date, most zolbetuximab studies have employed IHC (CLAUDETECT 18.2VR kit) to assess CLDN18.2 expression in patients with GC. However, given that 92% of the protein sequence of CLDN18.1 is consistent with CLDN18.2, the search for more specific anti-CLDN18.2 antibodies remains a major challenge [38]. An alternative ultrasensitive detection method to detect circulating tumor cellular CLDN18.2 RNA based on a molecular beacon demonstrated promising potential [66]. Furthermore, studies in patients from Korea and Japan revealed that 74.4 and 87% of patients had positive expression, and in these populations, 29.4 and 40% of patients had high expression levels, respectively. The positive criteria may be a main reason that the positive rates for CLDN18.2 are quite different between different studies in patients with GC. Standardization of the criteria determining positivity may be of interest in future studies. Regarding the difference in CLDN18.2 expression rates between different populations, such as Asians versus Caucasians, more investigations should be performed since the prevalence, incidence and mortality rates of GC in Western and Asian regions among these populations are quite different [67–70].

### Therapeutic agents targeting claudin18.2 for cancer immunotherapy

Different therapeutic agents targeting CLDN18.2 for cancer immunotherapy have been developed. The CLDN18.2-targeting therapeutic agents for clinical application include mAbs, bispecific antibodies (BsAbs), chimeric antigen receptor T (CAR-T) cells redirected to target CLDN18.2, and antibody–drug conjugates (ADCs). Regarding clinical trials related to CLDN18.2, as of the end of January 2022, there were 15 ongoing trials according to the chinadrugtrials.gov (NMPA) website and 28 trials on the clinicaltrials.gov (NCT) website, including 4 phase III, 4 phase II, 8 phase I/II and 27 phase I clinical trials. Most of these trials focus on solid tumors, including advanced unresectable GC, GEJ cancer, metastatic esophageal cancer, and PC. In terms of the types of agents, 22 trials employed mAbs, 3 trials employed bispecific mAbs, 9 trials employed CAR-T cells, and 9 trials employed ADCs. Some therapeutic agents were registered in both the NCT and NMPA platforms (Table 1). Here, we summarize recent advances in some of the CLDN18.2-targeted agents developed and their clinical applications.

**Table 1:** Clinical trials of therapeutic agents registered in the NCT and NMPA platforms

Platform	Type of Agents	Agent	Organization	NCT Number	Status	Conditions	Phase	Enrollment	Start Dates
**Clinical trials registered in the NMPA platform**	mAb	Zolbetuximab (IMAB362)	Astellas Pharma	CTR20190258	Recruiting	CLDN18.2+, HER2- advanced/metastatic GC	III	550	4/19/2019
	mAb	Zolbetuximab (IMAB362)	Astellas Pharma	CTR20190261	Recruiting	CLDN18.2+, HER2- advanced/metastatic GC	III	500	4/19/2019
	mAb	AB011	CARsgen Therapeutics	CTR20200515	Recruiting	CLDN18.2+ solid tumors	I	103	5/21/2020
	mAb	TST001	Mabspace Biosciences	CTR20201281	Recruiting	Advanced/metastatic solid tumors	I	210	8/3/2020
	mAb	MIL93	Mabworks Biotech	CTR20202436	Recruiting	Advanced/metastatic solid tumors	I	228	12/2/2020
	mAb	M108	FutureGen Biopharm	CTR20210508	Recruiting	Advanced solid tumor, GC, ST	I	160	3/31/2021
	mAb	LM-102	LaNova Medicines	CTR20211708	Recruiting	CLDN18.2+ solid tumors	I	265	7/22/2021
	mAb	LM-302	LaNova Medicines	CTR20212820	Recruiting	Solid tumors	I	128	11/17/2021
	mAb	NBL-015	NovaRock Biotherapeutics	CTR20213166	Active, not recruiting	PC, ST	I	N/A	12/8/2021
	Bispecific antibody	Q-1802	Qurebio	CTR20210800	Recruiting	Advanced/metastatic solid tumors	I	66	4/14/2021
	CAR-T cell	CT041	Shanghai Keji Pharma	CTR20201940	Recruiting	CLDN18.2+, HER2- advanced/metastatic GC	II	102	10/9/2020
	ADC	ASKB589	Jiangsu Aosaikang	CTR20202121	Recruiting	Advanced solid tumors	II	214	10/29/2020
	ADC	CMG901	Keymed Biosciences	CTR20202456	Recruiting	Advanced/metastatic solid tumors	I	122	12/9/2020
	ADC	SYSA1801	CSPC Pharma	CTR20211879	Recruiting	Advanced/metastatic solid tumors	I	272	6/16/2021
	ADC	RC118	RemeGen	CTR20212857	Recruiting	Solid tumors	I/IIa	135	11/29/2021
**Clinical trials registered in the NCT platform**	mAb	Zolbetuximab (IMAB362)	Astellas Pharma	NCT03504397	Recruiting	Advanced unresectable GEJ cancer or GC	III	550	6/21/2018
	mAb	Zolbetuximab (IMAB362)	Astellas Pharma	NCT03505320	Active, not recruiting	Advanced unresectable GEJ cancer or GC	II	116	2018-0-69-29
	mAb	Zolbetuximab (IMAB362)	Astellas Pharma	NCT03653507	Recruiting	Advanced unresectable GEJ cancer or GC	III	500	26-Sep-18
	mAb	Zolbetuximab (IMAB362)	Astellas Pharma	NCT03816163	Recruiting	Metastatic PC	II	369	15-Mar-19
	mAb	AB011	CARsgen Therapeutics	NCT04400383	Recruiting	Solid tumor, GC, PC	I	103	4-Jun-20
	mAb	TST001	Mabspace Biosciences	NCT04495296	Recruiting	Advanced cancers	I	210	13-Aug-20
	mAb	ASKB589	Jiangsu Aosaikang Pharmaceutical	NCT04632108	Active, not recruiting	PC, solid tumors, ST	I, II	N/A	28-Jan-21
	mAb	BNT141–01	BioNTech SE	NCT04683939	Recruiting	Solid tumors	I, II	48	Dec-21
	mAb	LM102	LaNova Medicines	NCT04735796	Recruiting	Advanced solid tumors	I	30	16-Jun-21
	mAb	LS-CLDN18.2001	Mabspace Biosciences	NCT04989010	Active, not recruiting	Solid tumors	I	20	1-Aug-21
	mAb	LM-102	LaNova Medicines	NCT05008445	Recruiting	Advanced solid tumors	I, II	265	6-Oct-21
	mAb	ZL-1211	Zai Biopharmaceutical.	NCT05065710	Recruiting	Advanced solid tumors	I, II	162	Oct-21
	mAb	FL-301	Nanjing Kaedi Biotech, Flame Biosciences	NCT05181865	Active, not recruiting	Solid tumors	I, II	115	Feb-22
	Bispecific antibody	AMG-910	Amgen	NCT04260191	Active, not recruiting	GC or GEJ adenocarcinoma	I	16	29-Jun-20
	Bispecific antibody	Q-1802	Qurebio	NCT04856150	Recruiting	Esophageal tumors, PC, solid tumors, ST	I	66	4/14/2021
	CAR-T cell	CT041	CARsgen Therapeutics	NCT03159819	Recruiting	Advanced GC, PC	I	24	1-Apr-17
	CAR-T cell	Engineered CAR-T cells	Hunan Zhaotai Yongren Medical	NCT03198052	Recruiting	Lung cancer	I	30	1-Jul-17
	CAR-T cell	CT041	CARsgen Therapeutics	NCT03874897	Recruiting	Advanced solid tumors	I	123	26-Mar-19
	CAR-T cell	CT041	CARsgen Therapeutics	NCT04404595	Recruiting	GC, PC	I	30	23-Oct-20
	CAR-T cell	LCARC18S	Nanjing Legend Biotech Co.	NCT04467853	Recruiting	GC	I	34	21-Sep-20
	CAR-T cell	CT041	CARsgen Therapeutics	NCT04581473	Recruiting	GC, PC, GEJ adenocarcinoma	I, II	102	23-Oct-20
	CAR-T cell	LY011	Shanghai Longyao Biotechnology	NCT04966143	Recruiting	PC	I	30	1-Aug-21
	CAR-T cell	LY011	Shanghai Longyao Biotechnology	NCT04977193	Recruiting	Advanced gastric adenocarcinoma	I	18	1-Nov-21
	ADC	CMG901	KeyMed Biosciences	NCT04805307	Recruiting	Advanced solid tumors, ST	I	162/122	12/24/2020
	ADC	124I-18B10 (10 L) PET/CT	Transcenta	NCT04883970	Recruiting	GC	I	15	13-May-21
	ADC	SYSA1801	CSPC ZhongQi Pharma	NCT05009966	Recruiting	Advanced solid tumors, GC, GEJ cancer, PC	I	272	16-Sep-21
	ADC	CPO102-US-1001	Conjupro Biotherapeutics	NCT05043987	Active, not recruiting	PC, GC	I	72	15-Feb-22
	ADC	LM-302	LaNova Medicines	NCT05161390	Active, not recruiting	Advanced solid tumors	I, II	128	30-Dec-21

Abbreviations: *GEJ*, Gastroesophageal junction, *GC* Gastric cancer, *PC* Pancreatic cancer, *ST* Stomach tumor

### Monoclonal antibodies targeting claudin18.2

#### Zolbetuximab

Zolbetuximab (claudiximab, previously IMAB362) is a mouse chimeric mAb with a human IgG1 constant region that specifically binds to CLDN18.2 [71]. It can be expressed by Chinese hamster ovary cells after gene recombination. Zolbetuximab can specifically recognize and bind the CLDN18.2 protein with high affinity via its first extracellular domain without cross-binding to any other claudin family members [53, 72]. The estimated lower limit of detection of the ELISA for zolbetuximab was as low as 0.3 ng/ml [72]. Zolbetuximab has been shown to be highly selective for CLDN18.2 both in vivo and in vitro*,* binds to cancer-specific targets that are predominantly expressed in tumor cells and shows little or no binding in healthy tissues. It mediated effective and target-selective antibody-dependent cell-mediated cytotoxicity (ADCC) against GC cell lines with CLDN18.2 expression and induced complement-dependent cytotoxicity (CDC)-mediated lysis of CLDN18.2-expressing tumor cells in GC [73]. This unique cancer-cell selectivity allows for maximal anticancer potency with diminished toxicity, which broadens the therapeutic window and allows optimal dosing.

Zolbetuximab can also induce CLDN18.2 protein-positive PC cell apoptosis in vitro, and the amplitude of ADCC and CDC is directly correlated with cell surface CLDN18.2 levels [15]. To examine if gemcitabine, through upregulating CLDN18.2 expression, may have an augmenting effect on zolbetuximab-induced ADCC and EC50 values, the PC cell line DNA-G cells were pretreated with gemcitabine before ADCC assay was performed. The results showed that zolbetuximab-induced ADCC was more efficient in the gemcitabine-pretreated cells with lower EC50. The chemotherapeutic agent gemcitabine upregulated CLDN18.2 expression in cultured human PC cells and enhanced zolbetuximab-induced ADCC [15]. However, the exact mechanism by which zolbetuximab inhibits tumor cell proliferation and apoptosis is unclear. Zolbetuximab also slowed tumor growth, benefited survival, and attenuated metastasis development in both preclinical and phase I clinical studies [15, 74]. In a preclinical study, zolbetuximab exhibited in vivo antitumor activity in mouse xenograft models, and zolbetuximab alone and in combination with gemcitabine prevented lung metastasis formation in intravenous mouse xenograft models [15]. However, a phase I study in 15 patients with advanced GC or GEJ cancer demonstrated that although most patients showed progressive disease at weeks 4–5 after a single intravenous zolbetuximab infusion, one patient in the 600 mg/m2 dose group achieved and maintained stable disease for approximately 2 months [74].

In the NCT01630083 study, which included 161 advanced/recurrent gastric and GEJ cancer patients with CLDN18.2 expression ≥2+ in ≥40% of tumor cells who were not eligible for trastuzumab therapy, patients were randomized 1:1 to receive first-line EOX (epirubicin 50 mg/m2 and oxaliplatin 130 mg/m2 d1, and capecitabine 625 mg/m2 bid, d1–21; qd22) with or without zolbetuximab (loading dose 800 mg/m2, then 600 mg/m2 d1, qd21). The results showed that zolbetuximab combined with first-line chemotherapy induced a clinically relevant progression-free survival (PFS) and overall survival (OS) benefit and had a favorable risk/benefit profile. Zolbetuximab plus EOX improved PFS (mPFS 4.8 vs. 7.9 months; HR 0.47; *p* < .001) and OS (mOS 8.4 vs. 13.2 months; HR 0.51; p < .001) compared to EOX alone. Furthermore, in the subpopulation with very high CLDN18.2 expression (≥ 2+ intensity in ≥70% tumor cells), efficacy was more pronounced (mOS 9 vs. 16.7 mo; HR 0.45; p < .001). The most common zolbetuximab-related adverse events included vomiting, neutropenia, and anemia, which were mostly NCI-CTC grade 1/2. Grade 3/4 events were not significantly increased by zolbetuximab [67]. Other studies have shown that when combined with first-line chemotherapy drugs, zolbetuximab can also enhance T-cell infiltration and induce the release of inflammatory cytokines, significantly enhancing the curative effect [73, 75].

Clinical studies have confirmed the safety and efficacy of zolbetuximab in patients with CLDN18.2-positive tumors [11, 74]. A phase I clinical study of patients with CLDN18.2-positive gastroesophageal adenocarcinoma [76] reported that zolbetuximab achieved satisfactory results when used in combination with IL-2 and zoledronic acid. Of 20 evaluable patients, 11 achieved disease control, including 1 patient with partial response and 10 patients with stable disease. The median PFS was 12.7 weeks, and the median OS was 40 weeks. The common treatment-related adverse events were mostly grade 1 ~ 3 and included nausea and vomiting.

A randomized phase II study of zolbetuximab plus epirubicin, oxaliplatin, and capecitabine (Xeloda) (EOX) versus EOX alone for first-line treatment of advanced CLDN18.2-positive GC and GEJ adenocarcinoma patients (the FAST study) revealed that both progression-free survival (PFS) [hazard ratio (HR) = 0.44; 95% confidence interval (CI), 0.29–0.67; *P* < 0.0005] and OS (HR = 0.55; 95% CI, 0.39–0.77; P < 0.0005) were significantly improved with zolbetuximab + EOX compared with EOX alone. This significant PFS benefit was retained in patients with moderate-to-strong CLDN18.2 expression in ≥70% of tumor cells (HR = 0.38; 95% CI, 0.23–0.62; P < 0.0005). Significant improvement in PFS was also reported in the overall population [11].

Another multicenter, phase IIa study (NCT01197885) of zolbetuximab as a single agent in 54 patients with recurrent or refractory advanced adenocarcinoma of the stomach or lower esophagus (the MONO study) showed that among the 43 patients with antitumor activity data available, 4 achieved PR (9%), and 6 (14%) had SD, resulting in a clinical benefit rate of 23%. In a subgroup of patients with moderate-to-high CLDN18.2 expression in ≥70% of tumor cells, the overall response rate (ORR) was 14% (*n* = 4/29). Treatment-related adverse events occurred in 81.5% (*n* = 44/54) of patients; nausea (61%), vomiting (50%), and fatigue (22%) were the most frequent. These results indicated that zolbetuximab monotherapy was well tolerated and exhibited antitumor activity in patients with CLDN18.2-positive advanced GC or GEJ adenocarcinomas [65]. A phase III efficacy, safety and tolerability study of zolbetuximab plus mFOLFOX6 chemotherapy compared to placebo plus mFOLFOX6 as treatment for gastric and GEJ cancer is underway (NCT03504397) [77]. Alternatively, another phase III trial, GLOW (NCT03653507), comparing IMAB362 + CAPOX with placebo + CAPOX is also underway as a first-line treatment for patients with GEJ adenocarcinoma [78].

To date, zolbetuximab is the well-poised CLDN18.2-targeted treatment in clinical trials for patients with HER-2-positive advanced GC and has shown great potential to become another useful target in GC. To date, no therapeutic resistance associated with zolbetuximab has been observed. Although zolbetuximab was shown to be effective for patients with coexpression of CLDN18.2 and HER-2 in the FAST study [11], trials for patients with CLDN18.2-positive / HER-2-positive and CLDN18.2-positive / HER-2-negative disease are still ongoing. To determine whether zolbetuximab can be another targeted drug in addition to HER-2-targeted agents for GC therapy, more data are needed. Currently, the ongoing SPOTLIGHT study is underway to compare the effect of zolbetuximab with placebo in combination with chemotherapy as a first-line therapy in claudin-18.2-positive and HER-2-negative advanced gastric or GEJ cancer [79]. The advanced clinical trials of zolbetuximab with detailed trial data is summarized in Table 2.

**Table 2:** Summary of clinical trials of zolbetuximab

NCT/CTR Number	Status	Phase	Enrollment	Target indication population	Arms and interventions	Key inclusion criteria:	Outcome measures	Results summary
NCT01197885	Completed	II	54	International, multicenter, open-label, phase II study in patients with advanced adenocarcinoma of the stomach or the lower esophagus: the MONO study	Experimental: zolbetuximab Cohort 1 repeated doses of 300 mg/m2 Cohort 2 repeated doses of 600 mg/m2	Metastatic,R/R disease of advanced adenocarcinoma of the stomach or the lower esophagus proven by histology CLDN18.2 expression of the biopsy material from the cancer confirmed by IHC At least 1 measurable site of disease according to RECIST criteria	CR, PR	Among the 43 patients with antitumor activity data available, 4 achieved PR (9%), and 6 (14%) had SD, resulting in a clinical benefit rate of 23%. In the patients with CLDN18.2 expression in ≥70% of tumor cells, the ORR was 14% (n = 4/29) [65].
NCT01630083	Completed	II	252	First-line treatment of patients with CLDN18.2-positive advanced adenocarcinomas of the stomach, the esophagus or the GEJ	Active comparator: EOX treatment Experimental: EOX + zolbetuximab 800/600 mg/m2 Experimental: EOX + zolbetuximab 1000 mg/m2	• Histologically confirmed adenocarcinoma of the stomach, the esophagus or the GEJ • Inoperable locally advanced disease or recurrent or metastatic disease. • CLDN18.2 expression confirmed by IHC	PFS	Zolbetuximab plus EOX improved PFS (mPFS 4.8 vs. 7.9 months; HR 0.47; p < .001) and OS (mOS 8.4 vs. 13.2 months; HR 0.51; p < .001) compared to EOX alone. In the subpopulation with CLDN18.2 expression ≥2+ intensity in ≥70% tumor cells, efficacy was more pronounced (mOS 9 vs. 16.7 mo; HR 0.45; p < .001) [67].
NCT03653507 CTR20190261	Recruiting	III	500	First-line treatment of CLDN 18.2-positive, HER2-negative, locally advanced unresectable or metastatic gastric or GEJ adenocarcinoma: the FAST study	Experimental: Arm A (zolbetuximab plus CAPOX) Placebo comparator: Arm B (placebo plus CAPOX)	Histologically confirmed gastric or GEJ adenocarcinoma. Confirmed locally advanced unresectable or metastatic disease. CLDN18.2+ in ≥75% of tumor cells and HER2-negative	PFS	Both PFS [HR = 0.44; 95% CI, 0.29–0.67; P < 0.0005] and OS (HR = 0.55; 95% CI, 0.39–0.77; P < 0.0005) were significantly improved with zolbetuximab + EOX compared with EOX alone. PFS benefit was retained in patients with CLDN18.2 ≥ 70% of tumor cells (HR = 0.38; 95% CI, 0.23–0.62; P < 0.0005). Significant improvement in PFS in the overall population [11].
NCT03504397 CTR20190258	Recruiting	III	550	First-line treatment of CLDN18.2-positive, HER2-negative, locally advanced unresectable or metastatic gastric or GEJ adenocarcinoma	Experimental: Arm A (zolbetuximab plus mFOLFOX6) Placebo comparator: Arm B (Placebo plus mFOLFOX6)	• Histologically confirmed gastric or GEJ adenocarcinoma. • Locally advanced unresectable or metastatic disease。 • CLDN18.2+ in ≥75% of tumor cells and HER2-negative	PFS	Results are pending.

Abbreviations: *GEJ* Gastroesophageal junction, *GC* Gastric cancer, *PFS* Progression-free survival, *HR* Harzard ratio, *CI* Confidence interval, *R/R,* relapsed or refractory, *CR* Complete remission, *PR* Partial remission

#### TST001

TST001 is a novel humanized IgG1 mAb that specifically binds to cells expressing human CLDN18.2 with high affinity but not to cells expressing the closely related CLDN18.1. A preclinical study revealed that TST001 is comparable to zolbetuximab. TST001 loses fucosylation during cell culture and thus develops increased binding affinity for FcγRIIIa, which translates into more potent ADCC activity. At subnanomolar concentrations, TST001 showed ADCC activity against GC cells expressing medium to low CLDN18.2 levels in the presence of human PBMCs and NK cells, suggesting that it is significantly more potent than zolbetuximab. TST001 also showed more potent CDC and antibody-dependent cellular phagocytosis (ADCP) against CLDN18.2-expressing cells than zolbetuximab. In GC cell lines and patient-derived xenograft tumor models, TST001 showed more potent antitumor activity than zolbetuximab. Furthermore, the combination of TST001 with chemotherapeutic agents resulted in synergistic antitumor effects in these tumor models [80]. Currently, TST001 is being evaluated in phase I trials (NCT04396821 and NCT04495296) in both the US and China to assess its safety, tolerability, and antitumor activity in patients with advanced solid tumors, including but not limited to GC and PC.

Preliminary data from a phase I clinical study showed that TST001 alone or in combination with chemotherapies is safe, with more than forty patients evaluated. Promising antitumor activities were also observed in this study. In a TST001 monotherapy dose-escalation study, one of three late-line GC patients who were heavily pretreated with multiple lines of treatment, including chemotherapy, programmed cell death protein 1 (PD-1)-targeted immunotherapy, and anti-vascular endothelial growth factor (VEGF) inhibitors, achieved a partial response after 6 weeks of treatment. In addition, the patient also had rapid and significant tumor biomarker reduction post-TST001 treatment. In a TST001 plus capecitabine + oxaliplatin (CAPOX) combination study in first-line GC, a patient achieved partial response after 6 weeks of treatment. A phase IIa monotherapy study in CLDN18.2-expressing solid tumors, including GC, PC and other tumor types, will begin soon [81].

In another phase I clinical trial, 11 patients with advanced or metastatic solid tumors who progressed on or after standard treatments were treated with 3, 6, and 10 mg/kg TST001 Q3W. Nine patients were eligible for the DLT analysis, with no DLT reported, and the MTD was not reached. TST001 demonstrated a roughly linear PK profile, as both the Cmax and AUC increased proportionally across the dose range following the first dose. No drug accumulation was observed in the Q3W cohort. Furthermore, 10 mg/kg Q3W was designated as the RP2D for a further expansion study, and three additional patients were enrolled into the expansion phase at the 10 mg/kg Q3W dose. The most common AEs (> 20%) included nausea (64%), vomiting (50%), anemia (43%), hypoalbuminemia (29%), abdominal distension (21%), and constipation (21%). Five patients experienced grade 3 AEs, including increased blood pressure, increased conjugated bilirubin, hyponatremia, nausea and vomiting, and pulmonary embolism. Two patients experienced 3 SAEs, including hypoalbuminemia, jaundice, cholestasis, and pulmonary embolism. No treatment-related grade 4 or 5 event was reported. One patient with CLDN18.2-overexpressing gastric signet ring cell carcinoma who progressed on multiple lines of chemotherapies and anti-PD1 and anti-VEGF therapies in the 6 mg/kg cohort achieved a confirmed partial response at week 12. These results demonstrated that TST001 had a manageable and tolerable safety profile in patients with advanced solid tumors and preliminary antitumor activity in a heavily pretreated gastric cancer patient expressing CLDN18.2 [82]. Another open-label, multicenter, phase I clinical trial to evaluate the safety, maximum tolerated dose, pharmacokinetic (PK) profile and preliminary anticancer effects of TST001 in 23 patients with locally advanced or metastatic solid tumors (NCT04396821) is underway, with results pending [83].

Other mAbs have also been developed by different organizations and are in either preclinical or clinical trials. A list of these agents is summarized in Table 1. Moreover, new antibody drugs that are expected to have additional therapeutic effects have been developed by modifying existing antibody drugs. In one study, after humanized specific anti-CLDN18.2 single-chain fragment variable (scFv) mAbs were generated, CLDN18.2-specific CAR T cells were subsequently developed using the scFv as a targeting component, and this study will be discussed further in the CAR-T cells targeting CLDN18.2 section [84]. Furthermore, other mAb candidates, such as AB101, ASKB589 and DR30303, are also under evaluation in clinical trials, with more clinical research results pending (Table 1).

### Bispecific/trispecific antibodies targeting claudin18.2

Different studies have demonstrated that targeting CLDN18.2 with an ADC or bispecific agent could be a valid therapeutic approach for the treatment of GC and PC [85]. Clinical research focus has switched from novel mAbs to bispecific mAbs and trispecific mAbs. Bispecific mAbs and trispecific mAbs can guide T cells by binding to CD3 on T cells and targeting CLDN18.2 to improve T cell cytotoxicity with low toxicity. Different formats of novel agents have been developed in preclinical or early clinical trials to further improve the efficacy of CLDN18.2-targeted therapy. These therapies have proven superior to traditional therapy. Furthermore, a novel tetravalent bispecific (Tetrabi) antibody targeting CLDN18.2 has shown impressive antitumor activity and provides better efficacy than the bispecific form [86]. Tetrabi is a form of bispecific antibodies which were produced with scFv fragments of bispecific antibodies followed by site-directed pegylation to generate tetra-scFv fragments. Pegylated tetra-scFv exhibited cytotoxic effects in tumor cells, while their circulation time in blood significantly increased compared with monomeric antibody fragments [87]. Here, we summarize a couple of agents in clinical studies.

#### AMG-910

- AMG-910 is a half-life extended (HLE) BiTE (bispecific T-cell engager) antibody designed to engage CD3-positive T cells and CLDN18.2-expressing tumor cells. Through binding to CLDN18.2 on tumor cells and CD3 on T cells, AMG-910 redirects tumor cell lysis to kill tumor cells. At present, a phase I clinical study of patients with CLDN18.2-positive GC and GEJ adenocarcinoma is ongoing (NCT04260191). The indicated population for this trial is patients who are aged 18 years with histologically or cytologically confirmed metastatic or locally advanced unresectable CLDN18.2-positive GC/GEJ adenocarcinoma who have shown refractoriness to or have relapsed following 2 prior lines of therapy. Primary endpoints include dose-limiting toxicities and treatment-emergent and treatment-related adverse events. Secondary endpoints include the pharmacokinetics of AMG-910, objective response, duration of response, time to progression, 6-month and 1-year progression-free survival, and 1- and 2-year overall survival. Although there was a preliminary report about this trial at the European Society for Medical Oncology (ESMO) virtual congress in 2020 [88], the study is still being performed, and thus far, a final report has not been provided.

#### Q-1802

Q-1802 is a bispecific antibody targeting programmed death-ligand 1 (PD-L1) and CLDN18.2 It can bind to CLDN18.2 and mediate antibody-dependent cell-mediated cytotoxicity against tumor cells. On the other hand, the portion of the antibody recognizing PD-L1 blocks PD-1 signaling and activates innate immunity and adaptive immunity. Animal studies have shown that Q-1802 can accurately target tumor tissue and has strong efficacy in killing tumor cells, revealing a new therapeutic candidate for advanced solid tumors with CLDN18.2 expression [89]. A phase I clinical trial has been initiated in patients with advanced solid tumors (NCT04856150), with pending results.

Other efforts have been made to target CLDN18.2 with a CD3-targeting bispecific antibody and an antibody−drug conjugate, which are being tested in efficacy and preliminary toxicity studies. The anti-hCLDN18.2 ADC, CD3-targeting bispecific antibody and diabody, which targets a protein sequence conserved in rats, mice and monkeys, exhibited in vitro cytotoxicity in BxPC3/hCLDN18.2 (IC50 = 1.52, 2.03, and 0.86 nM) and KATO-III/hCLDN18.2 (IC50 = 1.60, 0.71, and 0.07 nM) cells, respectively, and inhibited the growth of pancreatic and gastric patient-derived xenograft tumors. In a rat exploratory toxicity study, the ADC was tolerated up to a dose of 10 mg/kg. In a preliminary assessment of tolerability, the anti-CLDN18.2 antibody (0.34 mg/kg) did not produce obvious signs of toxicity in the stomach of NSG mice 4 weeks after administration. This result indicates that targeting CLDN18.2 with an ADC or bispecific modality could be a valid therapeutic approach for the treatment of gastric and pancreatic cancer [85].

### CAR-T cells targeting CLDN18.2

Chimeric antigen receptor T (CAR-T)-cell therapy is based on engineering of T lymphocytes to express chimeric antigen receptors (CARs), which enables the modified T lymphocytes to recognize and respond to cancer cells independently of major histocompatibility complex (MHC) engagement. After in vitro proliferation, CAR-T cells are reinfused into the patient to drive antitumor immune responses [90, 91]. Different generations of CARs have been developed by modifying different parts of the CD3z chain, including the extracellular antigen-binding domain (usually the single chain variable fragment of an antibody), transmembrane domain, and intracellular signaling domain, in addition to modifying costimulators and/or elements responsible for cytokine secretion [90–93]. As CAR-T-cell technology has progressed and clinical success has been achieved using different CAR-T cells, the feasibility of scaling-up the production of allogeneic or universal CAR-T cells for clinical use as off-the-shelf CAR-T cells under good manufacturing practices has been explored [90, 94–96].

Studies have been performed to elucidate whether CAR-T cells redirected to target CLDN18.2 have the potential to be used as agents for the treatment of GC and other cancer types. After developing the CLDN18.2-specific single-chain variable fragment (scFv)-containing humanized antibodies hu8E5 and hu8E5-2I, CLDN18.2-specific CAR-T cells were developed using the scFvs as targeting moieties. Both hu8E5-28Z and hu8E5-2I-28Z CAR-T cells, comprising the CD28 costimulatory domain, potently suppressed tumor growth in a cancer cell line xenograft mouse model. Partial or complete tumor elimination was observed in CLDN18.2-positive GC PDX models treated with hu8E5-2I-28Z CAR-T cells, which persisted well in vivo and efficiently infiltrated into tumor tissues [84]. Therefore, CLDN18.2-specific CAR-T cells could be a promising treatment for GC and other CLDN18.2-positive tumors. To date, nine clinical trials with CAR-T-cell candidates have been initiated and are at different stages, and we summarize those trial available in open-access databases in Table 1. An ongoing phase I trial is exploring the therapeutic concentration and safety of CLDN18.2-targeting CAR-T-cell therapy in a larger population, assessing factors such as dose-limiting toxicity and the maximum tolerated dose (NCT03874897). More data need to be collected from clinical trials to provide more evidence of efficacy for clinical application. As the clinical trial cycle of CAR-T cells is much shorter than that of other drugs, if everything goes well, the CAR-T-cell products targeting CLDN18.2 could be approved by the FDA for clinical application in 2023.

#### CT041

CT041 is a candidate anti-CLDN18.2-targeted autologous CAR-T-cell product for the treatment of patients with CLDN18.2-positive solid tumors. Preliminary studies and an investigator-initiated study (CT041-CG4003 NCT03159819, NCT03874897) showed that CT041 had an acceptable safety profile and promising antitumor activities. Patients with CLDN18.2+ cancers of the digestive system received 2.5 × 10^8^, 3.75 × 10^8^ or 5 × 10^8^ CT041 CAR-T cells after preconditioning chemotherapy. In total, 37 patients with cancer of the digestive system, including 28 with GC, 5 with pancreatic ductal adenocarcinoma and 4 with other cancer types, received CT041 infusion and completed at least 12 weeks of follow-up after the first infusion for the last subject. With a median follow-up of 7.6 months (95% CI 5.6, 8.6), all patients experienced ≥G3 hematologic toxicity, while no dose-limiting toxicity (DLT) was observed. The ORRs for all patients and patients with GC were 48.6% (95% CI, 31.9, 65.6%) and 57.1% (95% CI, 37.2, 75.5), respectively. In 18 patients with GC who failed at least 2 prior lines of therapy, 8 of whom (44%) had received anti-PD-(L)1 antibody, the ORR, median PFS and overall survival (OS) at the 2.5 × 10^8^ dose were 61.1% (11/18), 5.4 months (95% CI, 2.6, NE) and 9.5 months (95% CI, 5.2, NE), respectively [97]. A key phase II clinical trial (NCT04581473) is currently ongoing in patients with GC/GEJ adenocarcinoma and PC [98]. The same company is developing next-generation CAR-T-cell therapy candidates that target CLDN18.2, such as KJ-C1807 (CT048), for clinical trials in patients with GC and PC according to the company website.

#### LY011

LY011 is an anti-CLDN18.2 third-generation CAR-T-cell product prepared from allogeneic T lymphocytes that have been genetically modified to target the tumor-associated antigen (TAA) CLDN18.2 by using lentivirus vector technology with potential immunostimulating and antineoplastic activities. Upon administration, LY011 specifically recognizes and binds to CLDN18.2-expressing tumor cells, resulting in specific cytotoxic T lymphocyte (CTL)-mediated killing of CLDN18.2-expressing tumor cells. Currently, there are two ongoing phase I clinical trials (NCT04966143 and NCT04977193) in CLDN18.2-positive patients with PC and advanced GC. Based on the report on January 31, 2021, 4 patients with advanced malignant solid tumors (3 patients with gastric cancer and 1 patient with pancreatic cancer) had completed the DLT observation phase and received the first drug treatment. According to RECIST 1.1 evaluation criteria, among the 4 subjects, the best curative effect was disease remission (PR) in 2 subjects and disease stability (SD) in 2 subjects. When the report was released, the total ORR was 50.0% (2/4), and the total disease control rate (DCR) was 100% (4/4). In the lowest dose group, 1 × 10^6^ CAR-T cells/kg, the objective remission rate was 66.67% (2/3), and the disease control rate was 100% (3/3). No serious adverse events or adverse events causing the subject to discontinue treatment occurred. Most of the reported adverse events were mild or moderate. Three of the four subjects experienced only grade I cytokine release syndrome (CRS)-related fever after CAR-T-cell reinfusion. No CAR-T-cell-associated encephalopathy syndrome (CRES), suspected unexpected serious adverse reactions (SUSARs) or unique safety problems were reported. These preliminary data show that LY011 is safe and well tolerated with good antitumor effects in subjects with advanced malignant solid tumors [99]. More clinical studies need to be performed.

#### LCAR-C18S

LCAR-C18S was developed by Nanjing Legend Biotech Co. as a CAR-T-cell therapy targeting CLDN18.2. It has entered a phase I clinical trial for the treatment of GC and PC (NCT04467853, also known as BM2L201910) to determine its safety, tolerability and efficacy [100]. In addition, more candidates have been developed by other companies and are in the preclinical or early clinical research phases with clinical study results pending.

### ADCs

Antibody–drug conjugates (ADCs) are a powerful class of anticancer therapeutic agents that can deliver highly cytotoxic molecules directly to cancer cells. ADCs have been used in many patients with solid tumors and hematological malignancies. To date, twelve ADCs have received FDA approval, with numerous others in different clinical research stages [101, 102]. ADCs consist of recombinant monoclonal antibodies (payloads) that are covalently bound to cytotoxic chemicals via synthetic linkers. The linkers have a key role in ADC outcomes because their characteristics substantially impact the therapeutic index, efficacy and pharmacokinetics of these drugs. The linkers used in ADC development can be classified as cleavable and noncleavable, and both have been proven to be safe in preclinical and clinical trials. Cleavable linkers have been used in many ADCs, such as hydrazone linkers in gemtuzumab ozogamicin targeting CD33 for patients with AML, dipeptide (VC) MMAE linkers in brentuximab vedotin (SGN-35) targeting CD30 for patients with Hodgkin lymphoma, and SMCC linkers in trastuzumab emtansine targeting HER2 for HER2-positive breast cancers [101, 102].

The cytotoxic drugs used as payloads are highly potent agents used to kill cancer cells. The microtubule inhibitors auristatins and maytansinoids are the drugs most frequently used for ADC development, in addition to the DNA synthesis inhibitors calicheamicin, doxorubicin and duocarmycins [101, 103].

As the most important component of ADCs, the antibody can specifically identify and bind to a well-characterized tumor antigen receptor and deliver the payload to the tumor cell in the process. Currently, Nectin4, CD79b, CD22, CD33, HER2, CD30, FOLR1, and TROP2 are the antigens most often targeted by ADCs. In addition, over 70 other antigens are in different stages of clinical development [101, 103–106]. CLDN18.2 is one such antigen that has recently attracted increasing attention.

Upon binding, the ADC is internalized by the tumor cell, where it undergoes lysosomal degradation, leading to the release of the cytotoxic drug. The targeted delivery of highly potent cytotoxic drugs aims to enhance antitumor activity while minimizing toxicity in normal tissues. Additionally, this therapeutic approach takes advantage of the favorable pharmacokinetic properties of antibodies to provide sustained delivery of the cytotoxic drug [102]. Although no ADCs targeting CLDN18.2 have been approved for clinical applications thus far, a few candidates have been developed with promising preliminary results, and some of them have entered clinical trials at different phases. Here, we summarize a few ADCs targeting CLDN18.2 in clinical trials.

#### CMG901

CMG901 is a CLDN 18.2-targeting ADC that consists of three components: a monoclonal antibody targeting CLDN18.2, a cleavable linker and a potent cytotoxic payload (MMAE). CMG901 binds to Claudin 18.2-positive cells via its monoclonal antibody portion. After binding, CMG901 is internalized into lysosomes by tumor cells and releases the cytotoxic payload, leading to cell cycle arrest and apoptosis of the tumor cells. A mechanism of action study showed that CMG901 can cause tumor cell death by several mechanisms: CMG901 can stimulate cellular and soluble immune effectors that activate antibody-dependent cellular cytotoxicity (ADCC) and complement-dependent cytotoxicity (CDC) to destroy Claudin 18.2-positive cells. Preclinical studies suggest that CMG901 can effectively kill gastric cancer cells with much stronger antitumor potency than a zolbetuximab analog or the CMG901 unconjugated antibody. CMG901 also showed good tolerability and a favorable safety profile in preclinical studies [107]. CMG-901 has been approved by the FDA for a phase I clinical trial in patients with GC/GEJ cancer (NCT04805307).

#### SYSA-1801

SYSA-1801 is a fully human mAb plus MMAE drug conjugate targeting CLDN18.2. The preclinical in vitro and in vivo animal experiments of SYSA-1801 showed that it can effectively target tumor cells through the anti-CLDN18.2 antibody and trigger endocytosis, causing the small molecule toxin MMAE to enter tumor cells to achieve antitumor effects. Preclinical studies have shown that SYSA1801 has excellent in vivo and in vitro activity and good safety for gastric cancer, pancreatic cancer and lung cancer, suggesting it is a promising agent with good efficacy in clinical trials. SYSA-1801 has been approved by both the FDA and CDE for clinical trials in the US and China (NCT05009966, CTR20211879), respectively, in patients with GC/GEJ cancer and PC with CLDN18.2 expression [108].

#### RC118

RC118, an ADC targeting Claudin18.2, has been used to treat locally advanced unresectable or metastatic malignant solid tumors. RC118 was first tested in patients in Australia in November 2021 in a phase I clinical trial [109] and has also entered phase I/II studies in the US (NCT05205850). RC118 is composed of a recombinant humanized anti-CLDN18.2 monoclonal antibody and monomethyl auristatin E (MMAE), a potent tubulin binder with a half maximal inhibitory concentration (IC50) in the subnanomolar range, as the cytotoxic payload, and these factors are conjugated to each other through a cathepsin cleavable linker at an optimized drug–antibody ratio. RC118 is in a phase I clinical study as a treatment for CLDN18.2-positive patients with various types of solid tumors [110]. In addition, more candidates, such as CPO102-US-101 and LM-302, are under evaluation in clinical trials (Table 1), with more clinical research results pending.

### Challenges related to claudin18.2-targeted therapies

Due to the different detection methods, positive thresholds and patient populations used in different studies, the CLDN18.2 positive rate varies. One of the challenges in detection is that the detection method must be standardized by using a highly specific reagent to distinguish splice variants such as CLDN18.2 versus CLDN18.1. Thus, a unified detection method to specifically identify CLDN18.2 should be used [111]. Once the detection method is standardized, patient selection based on a CLDN18.2 positive threshold (for example, ≥ 75% tumor cells with intensity ≥2+) will enhance precision. Further research is necessary to standardize the optimal cutoff value used to determine CLDN18 positive expression for the identification of patients who should receive the targeted therapy. Whether different patient groups (races) need different cut-off expression levels needs to be further explored. Another controversy is the prognostic value of CLDN18.2. Direct evidence that CLDN18.2 can be used as a meaningful prognostic indicator similar to HER-2 is still lacking. Further research is needed to determine the prognostic value.

The development of a large spectrum of CLDN18.2 therapeutic drugs has been initiated in recent years. However, in terms of progress, most CLDN18.2 projects are still in the early clinical development stage. Among the candidates targeting CLDN18.2 for tumor immunotherapy, mAbs feature rich and mature technological development experience, effective commercial production strategies and reasonable cost, sound regulatory systems and a history of clinical use by doctors, and thus, these agents will undoubtedly be important in future treatments. Therefore, most of the CLDN18.2-targeted agents under development are mAbs. Bispecific/trispecific mAbs against CLDN18.2 are new and innovative drug formats, but they face great challenges in production, regulation and safety.

Theoretically, CLDN18.2 ADCs will have some advantages in avoiding the drug resistance of mAbs, but they also face common ADC-related obstacles. ADCs targeting CLDN18.2 have seen less clinical development and clinical application, and due to toxicities, future clinical development of CLDN18.2 ADCs should focus on important properties, which include mAbs with high binding affinity for the target antigen, minimal immunogenicity and a proper half-life for antitumour activity to avoid unwanted side effects, including off-target toxicity and premature elimination from circulation due to immunogenicity [112–114].

Although promising results have been achieved in different clinical applications, CAR-T-cell therapy has failed to have a large impact in the field of solid tumors, and the clinical therapeutic effects of CLDN18.2 CAR-T cells need to be further explored. The main reasons for the failure of CAR-T-cell therapy in the treatment of solid tumors include the heterogeneity of tumors and the different response rates of different patients to CAR-T-cell therapy; solid tumor microenvironments not only contain regulatory T cells (Tregs), tumor-associated macrophages (TAMs) and other immunosuppressive cells but also overexpress TGF-β, IL-10, IL-4 and other cytokines, which have immunosuppressive effects. These factors significantly reduce the efficacy of CAR-T cells. Therefore, avoiding immunosuppression in the microenvironment of solid tumors and maintaining the level of local CAR-T cells for a long time are challenging. As CAR-T cells are an individualized therapy, they have a complicated production process and high cost, which are other disadvantages with the use of CAR-T cells in the majority of patients. The emergence of universal CAR-T cells aims to solve these shortcomings, since this strategy can use lymphocytes from healthy donors to make the production process more reliable and easier. It also avoids the challenge of collecting high-quality T cells from patients due to lymphocytopenia caused by chemotherapy or severe disease. However, universal CAR-T cells may face GVHD challenges since they are of allogeneic origin.

At present, clinical applications mainly focus on the combination of targeted drugs and chemotherapy/immunotherapy. Although there is no report of its combination with immunotherapy, targeting CLDN18.2 can theoretically promote T-cell infiltration and antigen presentation and thus improve the efficacy of immune checkpoint inhibitors [7, 115]. Antiangiogenic drugs such as bevacizumab can activate IgG downstream effectors involved in ADCC, thus enhancing the effect of zolbetuximab. Chemotherapeutic agents such as gemcitabine can upregulate CLDN18.2 expression and enhance zolbetuximab-induced ADCC, therefore directly inducing cancer cell apoptosis [15]. In addition, chemotherapy makes tumor cells more sensitive to zolbetuximab by increasing the expression of CLDN18.2 and thus inducing proinflammatory cytokines [15]. Preclinical and clinical data show that zolbetuximab combined with chemotherapy can improve the survival rate of patients with CLDN18.2-positive advanced GC. However, some of the grade > 3 adverse effects observed in the FAST study, such as neutropenia (11.7%) and leukopenia (7.8%), from both chemotherapy and zolbetuximab could be great challenges during treatment [11]. Another factor that should be considered is the racial differences between Eastern and Western populations, which may have different responses to the same treatment regimen or strategies.

## Conclusion and outlook

With the deepening of research on the efficacy of targeted therapy and genotyping, targeted therapy has become a new choice for individualized and comprehensive treatment of malignant tumors. Targeted therapy can be combined with radiotherapy, chemotherapy and other strategies to improve the curative effect and reduce drug resistance. Due to its highly selective and stable expression in specific tumor tissues, CLDN18.2 has become a popular molecular target for antitumor drugs in recent years. Some studies have revealed very promising results of CLDN18.2-targeted drugs. Clinical research focus has switched from novel mAbs to bispecific mAbs and trispecific mAbs, in addition to other types of targeted therapies, such as CAR-T-cell therapy. In preclinical and early clinical studies, although BiTEs have achieved success in PDX mouse models, further confirmation of their effect and safety in humans is still needed. CLDN18.2-targeted monotherapy combined with other therapies, such as chemotherapy and CAR-T-cell therapy, has also been explored. However, CLDN18.2-targeted drugs still need to be further evaluated in clinical trials. Future CLDN18.2-targeted therapeutic research should also focus on the microenvironment and metabolic characteristics of tumor cells. Although no significant adverse events have been reported, grade 1 or 2 CAR-T-cell therapy-specific CRS has occurred, suggesting that cytokine storm may theoretically challenge clinical application. More clinical trials are required to explore the safety and efficacy of CLDN18.2 CAR-T-cell therapy in larger populations. These findings will provide more evidence of efficacy. Although no drug targeting CLDN18 has been approved for clinical application worldwide yet, global research and development have demonstrated that CLDN18.2 targeting candidates may become an important alternative for GC targeted treatment after HER-2-targeted agents. We believe that with continued research progress, new CLDN18.2-targeted drugs will improve diagnosis and treatment to benefit more patients with malignant tumors, such as GC/GEJ cancers.

## Data Availability

All clinical trials related information was obtained from public databases or from the related company’s announcement or website information.

## Abbreviations

3′-UTR: 3′-UTR: 3′-untranslated region
ADC: Antibody coupled drugs
ADCC: antibody-dependent cellular cytotoxicity
ADCP: Antibody-dependent cellular phagocytosis
BiTE: Bispecific T cell engager
BsAbs: Bispecific antibodies
CAPOX: Capecitabine (Xeloda) Oxaliplatin (Eloxatin)
CAR-T: Chimeric antigen receptor T
C-CPE: *Clostridium perfringens* enterotoxin
CDC: Complement-dependent cytotoxicity
CLDN18.2: CLDN18.2: Claudin18.2
CLDNs: CLDNs: Claudins
CREB: Cyclic AMP-responsive element binding protein
CTL: Cytotoxic T-lymphocyte
DLT: Dose-limiting toxicity
EGFR: Epidermal growth factor receptor
EOX: Epirubicin, oxaliplatin, capecitabine (Xeloda)
ERK: Extracellular signal-regulated kinase
ESMO: European society for medical oncology
GC: Gastric cancer
GEJ: Gastroesophageal junction
GEO: Gene Expression Omnibus
HLE BiTE: Half-life extended bispecific T cell engager
HPA HPA: Human Protein Atlas
HR: Hazard ratio
IPF: Idiopathic pulmonary fibrosis
LN: Lymph node
mAb: Monoclonal antibody
NPMA: National Medical Products Administration
NSCLC: Non-small-cell lung cancer
ORR: Overall response rate
OS: Overall survival
OS: Overall survival
PC: Pancreatic cancer
PD-1: Programmed cell death protein 1
PKA: Protein kinase A
RHOA: Ras homolog gene family member A
SRCC: Signet-ring cell carcinoma
TCGA: The Cancer Genome Atlas
TJs: Tight junctions
TMDs: Transmembrane domains
TTF-1: Thyroid transcription factor 1
